# Development of an ontology for laparoscopic transabdominal adrenalectomy via a comprehensive modified Delphi survey and its validation on a multicentric pilot data set for surgical training and future video analysis with machine learning algorithms

**DOI:** 10.1093/bjs/znae148

**Published:** 2024-06-25

**Authors:** Barbara Seeliger, Sofia Di Lorenzo, Pier F Alesina, Laurent Brunaud, Costanza Chiapponi, Carmela De Crea, Gianluca Donatini, Maurizio Iacobone, Özer Makay, Radu Mihai, Martina T Mogl, Didier Mutter, Nicolas Padoy, Fausto Palazzo, Oscar Vidal, Francesco Pennestrí, Jacques Marescaux, Michel Vix, Marco Raffaelli

**Affiliations:** Institute of Image-Guided Surgery, IHU Strasbourg, Strasbourg, France; Department of Digestive and Endocrine Surgery, University Hospitals of Strasbourg, Strasbourg, France; Research Institute Against Digestive Cancer, IRCAD, Strasbourg, France; ICube, UMR7357, CNRS, INSERM U1328 RODIN, Université de Strasbourg, Strasbourg, France; Division of Endocrine and Metabolic Surgery, Fondazione Policlinico Universitario Agostino Gemelli—IRCCS, Rome, Italy; Centro di Ricerca in Chirurgia delle Ghiandole Endocrine e dell'Obesità, Università Cattolica del Sacro Cuore, Rome, Italy; Department of Surgery, Division of Endocrine Surgery, Helios Universitätsklinikum Wuppertal, Universität Witten-Herdecke, Wuppertal, Germany; Unit of Endocrine and Metabolic Surgery, Department of Surgery (CVMC), CHU Nancy—Hospital Brabois Adultes, University of Lorraine, Nancy, France; Faculty of Medicine, INSERM NGERE/U1256 ‘Nutrition, Genetics, Environmental Risks’ University of Lorraine, Nancy, France; Department of General, Visceral, Cancer and Transplant Surgery, University Hospital Cologne, Cologne, Germany; Centro di Ricerca in Chirurgia delle Ghiandole Endocrine e dell'Obesità, Università Cattolica del Sacro Cuore, Rome, Italy; Division of Endocrine Surgery, Ospedale Isola Tiberina—Gemelli Isola, Rome, Italy; Department of General and Endocrine Surgery, Centre Hospitalier Universitaire de Poitiers, Poitiers, France; INSERM U1313-IRMETIST, Université de Poitiers, Poitiers, France; Endocrine Surgery Unit, Department of Surgery, Oncology, and Gastroenterology, University Hospital, University of Padua, Padua, Italy; Center for Endocrine Surgery, Ozel Saglik Hospital, Izmir, Turkey; Department of Endocrine Surgery, Churchill Cancer Centre, Oxford University Hospitals NHS Foundation Trust, Oxford, UK; Department of Surgery, CCM | CVK, Charité—Universitätsmedizin Berlin, corporate member of Freie Universität Berlin and Humboldt-Universität zu Berlin, Berlin, Germany; Institute of Image-Guided Surgery, IHU Strasbourg, Strasbourg, France; Department of Digestive and Endocrine Surgery, University Hospitals of Strasbourg, Strasbourg, France; Research Institute Against Digestive Cancer, IRCAD, Strasbourg, France; Institute of Image-Guided Surgery, IHU Strasbourg, Strasbourg, France; ICube, UMR7357, CNRS, INSERM U1328 RODIN, Université de Strasbourg, Strasbourg, France; Department of Endocrine Surgery, Hammersmith Hospital, Imperial College Healthcare NHS Trust, London, UK; Department of General and Endocrine Surgery, Institute of Digestive and Metabolic Diseases, Hospital Clínic Barcelona, Universitat de Barcelona, Barcelona, Spain; Division of Endocrine and Metabolic Surgery, Fondazione Policlinico Universitario Agostino Gemelli—IRCCS, Rome, Italy; Centro di Ricerca in Chirurgia delle Ghiandole Endocrine e dell'Obesità, Università Cattolica del Sacro Cuore, Rome, Italy; Research Institute Against Digestive Cancer, IRCAD, Strasbourg, France; Department of Digestive and Endocrine Surgery, University Hospitals of Strasbourg, Strasbourg, France; Research Institute Against Digestive Cancer, IRCAD, Strasbourg, France; Division of Endocrine and Metabolic Surgery, Fondazione Policlinico Universitario Agostino Gemelli—IRCCS, Rome, Italy; Centro di Ricerca in Chirurgia delle Ghiandole Endocrine e dell'Obesità, Università Cattolica del Sacro Cuore, Rome, Italy

Right and left transabdominal lateral adrenalectomy (RTLA/LTLA) are relatively rare procedures^1^, requiring appropriate training and experience, with a long learning curve^2^. Procedure-related complications can be serious, including liver, duodenal or inferior vena cava injury on the right side, or colonic, pancreatic, gastric, splenic and splenic vascular injury on the left. A video recording of the procedure provides documentation and may help identify and avoid causes of intraoperative complications. Meanwhile, structured video analysis can provide individual training and would pave the way for automatic workflow recognition and safety alerts via artificial intelligence (AI) systems^3^.

Laparoscopic and retroperitoneoscopic adrenalectomy are the ‘gold standard’ for most surgically treated adrenal diseases^4^. A surgical ontology, outlining the procedure via its hierarchical decomposition into different levels of granularity with a standardized vocabulary, is therefore required to provide a machine-readable process model as a prerequisite for AI analysis of the surgical workflow^5^. To address this, we developed an ontology for multicentre use and validated its application for standardized RTLA/LTLA video analysis in a clinical pilot.

An ontology was developed by a steering committee (BS/SDL/MR), hierarchically structuring RTLA/LTLA into five discrete phases—Preparation, Exposure, Dissection of the main adrenal vein, Dissection of the adrenal gland, Extraction and disassembling—with 27 RTLA and 25 LTLA steps (*Figs S1* and *S2*). For both RTLA and LTLA, a sixth phase named other intervention was added for cases of simultaneous procedures.

Senior academics (*n* = 17) from across Europe with >10 years of experience in adrenal surgery were invited to take part in a modified Delphi process across two rounds (*Fig. S3*). Panellist response rate was 88% in the first round (15/17) and 100% (15/15) in the second round. Consensus, defined as ≥80% agreement for each statement, was reached for LTLA in all 38 statements and for RTLA in 39/40 statements (97.5%; *Tables S1* and *S2*). The content analysis of the panellists’ comments identified the recurring theme of the identification of the right renal vein during RTLA as a facultative step rather than a mandatory one, depending on tumour and anatomical factors.

Two surgeons then applied the defined ontology to annotate each phase and step in 4 RTLA and 4 LTLA videos from two centres in a machine-readable format. Near-complete agreement between both surgeons was observed for the phase and step durations (*Fig. 1*). Video annotations revealed that completion of mandatory steps is required to proceed to the next phase, although the sequence of steps may be adapted to the individual anatomy. Procedure progression is not necessarily linear, as dissection may require interruption of a phase/step, and progress one step forward with completion of the previous step later. The Supplementary material to this letter describes the key phases and steps illustrated in *Video S1* (RTLA) and *Video S2* (LTLA) as they would appear in a dedicated video annotation software.

**Fig. 1:**
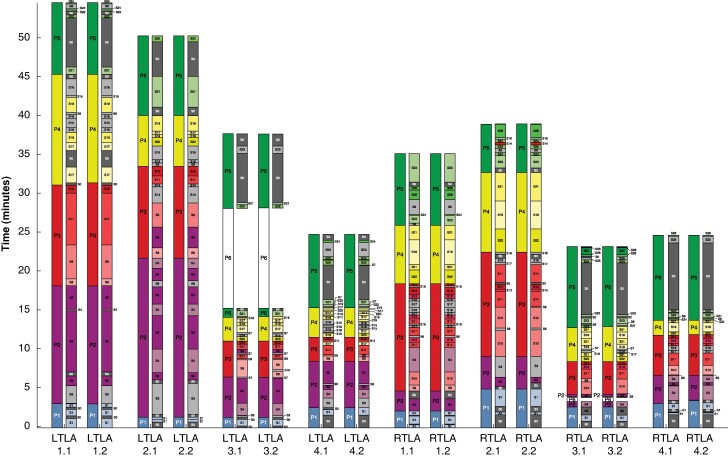
**Duration of the phases and steps in relation to the total duration of the video recordings of 4 LTLA and 4 RTLA from Strasbourg and Rome** Mandatory steps are coloured, facultative steps are grey. In case of P6 (Phase 6—Other intervention), no adrenalectomy steps are present. Each video was annotated by two surgeons. The inter-annotator agreement is illustrated by an almost complete match of phase and step durations for each video.

In this study, we have defined a novel consensus-derived ontology for RTLA and LTLA. The ontology is suitable for standardized and multicentric video assessments as a baseline for stepwise understanding, surgical training and video analysis. The ontology will aid machine learning algorithms for future AI applications such as automated phase and step recognition, surgical skill assessment, procedural training, and ultimately ‘real-time’ intraoperative guidance.

## Supplementary Material

znae148_Supplementary_Data

## Data Availability

Raw data are available on request, and upon approval of a data-sharing agreement and analysis plan.
