# Supplementary material for: Development of an ontology for laparoscopic transabdominal adrenalectomy via a comprehensive modified Delphi survey and its validation on a multicentric pilot data set for surgical training and future video analysis with machine learning algorithms
Source: Br J Surg. 2024 Jun 25;111(6):znae148. doi: 10.1093/bjs/znae148 (PMC11196904; doi:10.1093/bjs/znae148)
Supplement: znae148_Supplementary_Data [file znae148_supplementary_data.zip › Supplementary-Material-BJS-final.docx]

**SUPPLEMENTARY MATERIALS**

**Development of an ontology for laparoscopic transabdominal adrenalectomy via a comprehensive modified Delphi survey and its validation on a multicentric pilot dataset for surgical training and future video analysis with machine learning algorithms**

Barbara Seeliger^1,2,3,4^*, Sofia Di Lorenzo^5,6^*, Pier F. Alesina^7^, Laurent Brunaud^8^, Costanza Chiapponi^9^, Carmela De Crea^6,10^, Gianluca Donatini^11,12^, Maurizio Iacobone^13^, Özer Makay^14^, Radu Mihai^15^, Martina T. Mogl^16^, Didier Mutter^1,2,3^, Nicolas Padoy^1,4^, Fausto Palazzo^17^, Oscar Vidal^18^, Francesco Pennestrí^5,6^, Jacques Marescaux^3^, Michel Vix^2,3^, Marco Raffaelli^5,6^

**Supplementary Materials - Index**

| **Supplementary Methods** | *page 2* |
| --- | --- |
| Development of the TLA ontologies for right and left adrenalectomy | *page 2* |
| Modified Delphi consensus | *page 2* |
| Validation of the TLA ontologies for right and left adrenalectomy on a pilot series of anonymised adrenalectomy videos | *page 3* |
| **Supplementary Results** | *page 3* |
| Table S1: RTLA consensus | *page 6* |
| Table S2: LTLA consensus | *page 8* |
| **Supplementary Discussion** | *page 3* |
| **Supplementary Figures and Videos** |  |
| Figure legends | *page 5* |
| **References** | *page 10* |

**Supplementary Methods**

The study was carried out in three phases: (I) Development of the TLA ontologies for right and left adrenalectomy, (II) Modified Delphi consensus and (III) Validation of the TLA ontologies for right and left adrenalectomy on a pilot series of anonymized adrenalectomy, developed and led by a steering committee of 3 endocrine surgeons (BS, SDL, MR).

**Development of the TLA ontologies for right and left adrenalectomy**

Based on a textbook framework^1^ and the standardised operative technique for right and left TLA at the steering committee’s departments, the procedures were hierarchically broken down into phases and steps following the ontological approach reported in the Society of Gastrointestinal and Endoscopic Surgeons (SAGES) consensus recommendations on annotation frameworks for surgical videos^2, 3^**(Fig.S1/S2)**. The present ontologies aimed at the assessment of the surgical workflow that is exclusively visible on laparoscopic video recordings, for manual or automated analysis with Artificial Intelligence (AI) algorithms.

*Temporal annotations of operations:* The first temporal component are *phases*, structured into Access, Execution of surgical objectives, and Closure. The second temporal component are *steps*, characteristic and essential segments of a procedure. Steps are either completed at once, or interrupted to return to a previous step, and some are applicable for several procedures. *Null step* (step 0) represents an idle time with no surgical action visible within the laparoscope’s field of view (e.g., when instrument preparations are occurring outside the body cavity) and is defined to mark frames for exclusion that are unsuitable for activation of a recognition-prediction cycle by AI algorithms, to optimise machine learning training. It can occur during any phase and is always applied when the laparoscope is removed from the abdominal cavity.

*Mandatory* signifies that a given phase/step is compulsory to complete the surgical procedure.

*Facultative* signifies that a given phase/step is optional as it may or may not be performed depending on intraoperative circumstances.

Consequently, the proposed ontology contained definitions of 5 phases and 27 steps for RTLA **(Fig. S1)**, 5 phases and 25 steps for LTLA **(Fig. S2)**, and *Phase 6-Other Intervention* for both.

**Modified Delphi consensus**

Study design: For validation of the 39 RTLA and 37 LTLA ontology statements, a modified Delphi consensus technique was applied via two rounds of online surveys integrated into online questionnaires using a blinded electronic voting platform (Jotform, USA). The survey was conducted in October 2023 with one-week periods of response acquisition and analysis, respectively, for each of the two rounds. A four-point Likert scale was used for participants to rate each statement. A supermajority rule was used to define consensus (≥80% agreement) for each statement, and experts were asked to use the free-text boxes to go into detail regarding their reasoning and propose amendments in case of disagreements. Agreement of 100% was not re-subjected to a second survey round. Survey data collection and analysis with respect of European data protection regulations was ensured, and all participants chose to be named as a contributor.

The appropriate selection of a group of experts with a high and concordant skill level is critical to promote robustness and data validity in Delphi processes^4^. Consensus depends rather on group dynamics than on sample size and statistical power, and 10–18 experts are generally recommended on a Delphi panel^5, 6^. An international panel was thus constituted by inviting 17 senior academics with over 10 years of clinical experience in adrenal surgery across Europe **(Fig. S3)**, and most of these endocrine surgeons (n=14) were ESES members.

**Validation of the TLA ontologies for right and left adrenalectomy on a pilot series of anonymised adrenalectomy videos**

According to the General Data Protection Regulation, the principles of data protection do not apply to personal data rendered anonymous so that the subject is not or no longer identifiable. Anonymisation allows the reuse of data without infringing on people's privacy and storage of these data without limitation. Additionally, ethical committee approval was obtained (ID 6504, protocol N. 0000544/24, Comitato Etico Territoriale Lazio Area 3, Italy). The video recordings were archived in an anonymised format, with blackening of the times when the laparoscope was extracted from the abdominal cavity, to ensure privacy of patient and staff. There were no metadata other than the surgeon’s name and year of the procedure, and all surgeons consented to the analysis of their procedures as co-authors in the present study.

Uncut laparoscopic TLA videos recorded as part of routine surgical care were retrieved from the institutional archives. Two surgeons independently annotated the videos, documenting the timepoints for each phase and step duration for 4 RTLA and 4 LTLA videos from 2 centres in an Excel spreadsheet, as a pilot proof of concept for descriptive analysis. The RTLA and LTLA phases and steps durations were illustrated using the open-source Python language and matplotlib library (<https://pypi.org/project/matplotlib/>) for figures, and were overlaid onto the videos using the open source OpenCV library (<https://pypi.org/project/opencv-python/>).

**Supplementary Results**

In Round 1, 37/39 (95%) RTLA statements obtained consensus and 39/40 (97.5%) in Round 2, with one new statement in Phase 4 (Phase 1, 5/5; Phase 2, 8/8; Phase 3, 13/14; Phase 4, 6/6; Phase 5, 6/6; Phase 6, 1/1), as detailed in **Table S1**.

Consensus was obtained for 37/37 (100%) LTLA statements in Round 1 and for 38/38 (100%) in Round 2, with one new statement in Phase 4 (Phase 1, 5/5; Phase 2, 8/8; Phase 3, 12/12; Phase 4, 6/6; Phase 5, 6/6; Phase 6, 1/1), as detailed in **Table S2**.

**Supplementary Discussion**

Ontologies for laparoscopic procedures provide surgical process models in a computer-understandable format, providing data labelled with the information that AI systems learn to predict^7^. Nowadays, automated phase recognition is most developed for short operations with highly standardised workflows, such as laparoscopic cholecystectomy, and is emerging for more complex and oncologic procedures, such as laparoscopic distal gastrectomy and robotic colorectal surgery^8-10^.

Laparoscopic adrenalectomy is a demanding procedure requiring an often-protracted learning curve^11^. Despite the technical standardisation after more than two decades of minimally invasive adrenal surgery, there is still a risk of severe complications^12^. Laparoscopic adrenalectomy is thus a suitable candidate for detailed procedural analysis to support surgical and machine learning training with the aim to promote textbook outcomes in adrenal surgery.

Dissection of the main adrenal vein (Phase 3) is the crucial phase in both right and left laparoscopic adrenalectomy^12^ and panellists agreed that it should be performed, whenever possible, prior to any manipulation or mobilisation of the adrenal gland (Phase 4). These findings are in line with the literature, a no-touch technique is recommended particularly in cases of known or suspected adrenocortical carcinoma (ACC) and pheochromocytomas to avoid spillage of neoplastic cells and catecholamine release or the later occurrence of pheochromocytomatosis^13, 14^. According to the panel, an exception can be made for large tumours, as mobilisation of the adrenal gland may be necessary for exposure if the adrenal vein is initially difficult to access, still taking oncological principles into account. LTLA might be considered more technically demanding than RTLA due to the *en bloc* mobilisation of the spleen and pancreatic tail, and the position of the main adrenal vein within the perinephric adipose tissue^12^.

This study addressed total adrenalectomy, which is more linear than cortical-sparing approaches, in which some steps, such as division of the main adrenal vein, may be unnecessary. Specific steps taking the higher variability in partial adrenalectomy into account have to be complemented along with the ontology translation to retroperitoneoscopic and robotic approaches. Another limitation of this study may be the relatively restricted number of panellists. However, there is no gold standard sample size for Delphi panels, and reliable outcomes were reported with a small Delphi panel of similarly trained experts^15^. The number of 15 panellists was considered satisfactory as several Delphi studies were conducted with less than 15 experts^16^, and a reported threshold of 12^17^. Moreover, only high-profile professionals selected via strict inclusion criteria were invited, and the 88% response rate was above the 70% expected from literature reports^18^. Since all respondents completed both RTLA and LTLA surveys, there was no need to expand the panel. Furthermore, to minimise bias from a small sample size, the agreement threshold was set at 80%, above the usually reported 70-75%^19^.

Consensus was reached for all but one statement that concerned identification of the right renal vein in RTLA; 27% of panellists stated that this step should be optional instead of mandatory. In contrast to the obligatory identification of the left renal vein at its junction with the left main adrenal vein, identification of the right renal vein is not required for safe identification of the right main adrenal vein, which usually drains into the IVC 3–5 cm above the renal vein^20^. Although variants in (ad)renal venous anatomy are rare, they are described more often on the right side, and usually associated with adrenal tumour size >5cm and phaeochromocytomas^21^. Hence, the RTLA step 12 was not considered as mandatory, but strongly recommended in patients at higher risk for anatomical variants.

When applying the ontologies to a multicentric clinical pilot of 4 RTLA and 4 LTLA videos, agreement was observed for most of the phase and step durations annotated by two surgeons (Fig 1). For more fine-grained annotation instructions, items were highlighted such as creation of an additional step for insertion of foreign material into the abdominal cavity (e.g., a swab for retraction or haemostasis), or separate labels for the repositioning of the spleen and pancreatic tail as well as the colon at the end of LTLA. Additionally, the steps S15/S16 were perceived as important to highlight from a didactic point of view, differentiating inferior, medial and superior arteries. For unforeseen events, a step “other” could be added to document e.g. divisions of accessory structures. Procedural progress is not necessarily chronological/linear, as dissection can require moving one step forward or to perform partial steps and later completion according to the individual anatomy of the patient and the neoplastic lesion.

Procedural phases and steps are the basic building blocks for performing state-of-the-art surgical procedures. In robotic surgery in particular, modular training with the trainee performing partial procedural steps was recommended for gradual progress towards safe performance of complete procedures^22^. Operative video recording and analysis is crucial for teaching expert techniques to surgical trainees. It paves the way for continuous surgical education, video-based assessment for surgical accreditation, and holds considerable potential for eliminating substandard techniques which result in diverse outcomes and quality of life^23^. Since the first augmented-reality-assisted laparoscopic adrenalectomy^24^, guidance based on image analysis has been further developed as the principal axis for progress in minimally invasive surgery^23^. Ontologies for laparoscopic procedures provide surgical process models in a computer-understandable format, providing data labelled with the information that AI systems learn to predict^7^. Such systems need to activate a recognition-prediction cycle of the surgical workflow that should mimic expert surgeons’ cognitive processes: first, recognize the current situation at any time; then, predict the subsequent surgical activities^25^.

The next step is to apply the present ontologies to a multi-centre video dataset and extend it with identification of potential, observed, and critical errors to accurately characterise and promote standard approaches to RTLA and LTLA also applicable to partial adrenalectomy and robotic techniques. Such an approach was recently reported for the development of objective performance metrics for structured and quality-assured robot-assisted partial nephrectomy training^26^. Future studies should also analyse which steps and techniques influence the total length of an operation when comparing surgeons with different experience/workload. Phase and step durations must be correlated with clinical parameters to account for the impact of procedural difficulty and complications.

Preoperative imaging features such as tumour size, position and classification of the neoplastic lesion and the patient's BMI provide important information to enable the development of objective assessments based on multimodal analyses that are tailored to individual patient scenarios. This will allow to develop context-aware surgical guidance systems. In the new world of digital surgery, international surgical consortia will be able to make a decisive contribution to the education and training via computer-assisted adrenal surgery that should enable the best possible outcomes.

**Figure legends**

**Figure S1.** Hierarchical structure of phases and steps of the proposed laparoscopic Right Transabdominal Lateral Adrenalectomy (RTLA) ontology. Facultative items have a dashed border. In S0 null step, no significant activity potentially activating a recognition-prediction cycle by machine learning algorithms is recorded. Abdominal cavity exploration (S2) is not systematically performed beyond the area required for trocar placement. Adhaesiolysis (S3) is listed in Phase 2 but can already be required in Phase 1 for trocar positioning (S1). Sometimes, the triangular ligament is not as large, and its dissection (S4) can be skipped if the liver is mobile. Facultative mobilisation of the duodenum (S8) can be part of Phase 2 or 3.

**Figure S2.** Hierarchical structure of phases and steps of the proposed laparoscopic Left Transabdominal Lateral Adrenalectomy (LTLA) ontology. Facultative items have a dashed border. In S0 null step, no significant activity potentially activating a recognition-prediction cycle by machine learning algorithms is recorded. Abdominal cavity exploration (S2) is not systematically performed beyond the area required for trocar placement. Adhaesiolysis (S3) is listed in Phase 2 but can already be required in Phase 1 for trocar positioning (S1). Dissection of the left colonic flexure (S4) can be facultative according to individual anatomy. Dissection of the posterior and lateral aspects of the adrenal gland (S19) also includes medial dissection.

**Figure S3.** Geographic distribution of the European expert centres involved in the Delphi consensus.

**Supplementary videos**

**Video 1: Right total adrenalectomy – exemplary phases and steps Video_S1_still**

**Video 2: Left total adrenalectomy – exemplary phases and steps**

| **Statements** | **Round 1 (*n*=15)** | | | | | **Round 2 (*n*=15)** | | | | |
| --- | --- | --- | --- | --- | --- | --- | --- | --- | --- | --- |
|  | **SD** | **D** | **A** | **SA** | **A%** | **SD** | **D** | **A** | **SA** | **A%** |
| **Phase 1**  Do you agree that RTLA Phase 1 is "Preparation"? | 1 | 0 | 5 | 9 | 93.3 | 0 | 0 | 4 | 11 | 100 |
|  |  |  |  |  |  |  |  |  |  |  |
| Do you agree that Step 0 is "Null step" and that it is facultative? | 0 | 2 | 4 | 9 | 86.7 | 0 | 1 | 5 | 9 | 93.3 |
| Concerning the facultative step 0, do you agree that it can also be recorded in each subsequent phase of RTLA? | 0 | 3 | 4 | 8 | 80 | 0 | 0 | 6 | 9 | 100 |
| Do you agree that Step 1 is “Trocar positioning”? | 1 | 1 | 3 | 10 | 86.7 | 0 | 0 | 5 | 10 | 100 |
| Do you agree that Step 2 is “Abdominal cavity exploration”? | 0 | 0 | 5 | 10 | 100 | n/a | n/a | n/a | n/a | n/a |
| **Phase 2**  Do you agree that RTLA Phase 2 is “Exposure”? | 0 | 1 | 2 | 12 | 93.3 | 0 | 0 | 6 | 9 | 100 |
|  |  |  |  |  |  |  |  |  |  |  |
| Do you agree that Step 3 is “Adhaesiolysis” and that it is facultative? | 0 | 0 | 6 | 9 | 100 | n/a | n/a | n/a | n/a | n/a |
| Do you agree that Step 4 is “Dissection of the right triangular ligament (Round 1) / Dissection of the right triangular ligament and mobilization of the liver (Round 2)”? | 1 | 1 | 2 | 11 | 86.7 | 0 | 0 | 5 | 10 | 100 |
| Do you agree that Step 5 is “Superomedial retraction of the liver (Round1) / Superior retraction of the liver (Round 2)”? | 0 | 1 | 4 | 10 | 93.3 | 0 | 0 | 4 | 11 | 100 |
| Do you agree that Step 6 is “Haemostasis” and that it is facultative? | 0 | 1 | 4 | 10 | 93.3 | 0 | 1 | 6 | 8 | 93.3 |
| Concerning the facultative Step 6, do you agree that it can also be recorded in each subsequent phase of RTLA? | 0 | 0 | 8 | 7 | 100 | n/a | n/a | n/a | n/a | n/a |
| Do you agree that Step 7 is “Irrigation- aspiration” and that it is facultative? | 0 | 1 | 6 | 8 | 93.3 | 0 | 0 | 9 | 6 | 100 |
| Concerning the facultative Step 7, do you agree that it can also be recorded in each subsequent phase of RTLA? | 0 | 1 | 8 | 6 | 93.3 | 0 | 0 | 7 | 8 | 100 |
| **Phase 3**  Do you agree that RTLA Phase 3 is “Dissection of the main adrenal vein”? | 0 | 3 | 3 | 9 | 80 | 0 | 1 | 7 | 7 | 93.3 |
|  |  |  |  |  |  |  |  |  |  |  |
| Do you agree that Step 8 is “Mobilization of duodenum” and that it is facultative? | 1 | 1 | 8 | 5 | 86.7 | 0 | 2 | 6 | 7 | 86.7 |
| Do you agree that Step 9 is “Identification of the IVC”? | 1 | 0 | 3 | 11 | 93.3 | 0 | 2 | 3 | 10 | 86.7 |
| Do you agree that Step 10 is “Opening of the subhepatic peritoneum”? | 0 | 0 | 4 | 11 | 100 | n/a | n/a | n/a | n/a | n/a |
| Do you agree that Step 11 is “Dissection of the medial edge of the adrenal gland from the IVC”? | 0 | 1 | 4 | 10 | 93.3 | 0 | 0 | 7 | 8 | 100 |
| Do you agree that Step 12 is “Identification of the right renal vein”? | 0 | 4 | 4 | 7 | **73.3** | 0 | 4 | 6 | 5 | **73.3** |
| Do you agree that Step 13 is “Identification of the right main adrenal vein”? | 0 | 0 | 3 | 12 | 100 | n/a | n/a | n/a | n/a | n/a |
| Do you agree that Step 14 is “Ligation and division of the right adrenal vein (Round 1) / Sealing and division of the right adrenal vein (Round 2)”? | 0 | 0 | 3 | 12 | 100 | 0 | 0 | 5 | 10 | 100 |
| Do you agree that Step 15 is “Identification of an accessory adrenal vein and that it is facultative”? | 0 | 0 | 6 | 9 | 100 | n/a | n/a | n/a | n/a | n/a |
| Do you agree that Step 16 is “Ligation and division of the accessory adrenal vein” and that it is facultative (Round 1) / “Sealing and division of the accessory adrenal vein” and that it is facultative (Round 2)”? | 0 | 0 | 7 | 8 | 100 | 0 | 0 | 8 | 7 | 100 |
| Do you agree that Step 17 is “Identification of adrenal arteries” and that it is facultative? | 0 | 0 | 9 | 6 | 100 | n/a | n/a | n/a | n/a | n/a |
| Concerning the facultative Step 17, do you agree that it can also be recorded in the subsequent phase of RTLA? | 0 | 0 | 11 | 4 | 100 | n/a | n/a | n/a | n/a | n/a |
| Do you agree that Step 18 is “Ligation and division of adrenal arteries” and that it is facultative (Round 1) / Sealing and division of adrenal arteries” and that it is facultative (Round 2)?” | 0 | 2 | 9 | 4 | 86.7 | 0 | 0 | 9 | 6 | 100 |
| Concerning the facultative Step 18, do you agree that it can also be recorded in the subsequent phase of RTLA? | 0 | 0 | 11 | 4 | 100 | 0 | 0 | 8 | 7 | 100 |
| **Phase 4**  Do you agree that RTLA Phase 4 is “Dissection of adrenal gland”? | 0 | 1 | 5 | 9 | 93.3 | 0 | 0 | 9 | 6 | 100 |
|  |  |  |  |  |  |  |  |  |  |  |
| Do you agree that Step 19 is “Dissection of the adrenal gland from the upper pole of the kidney *en* *bloc* with periadrenal fat”? | 0 | 0 | 5 | 10 | 100 | n/a | n/a | n/a | n/a | n/a |
| Do you agree that Step 20 is “Identification and preservation of superior pole renal arteries” and that it is facultative (Round 1) / “Identification and preservation of superior pole renal arteries and that preservation of the vessels is compulsory, but dissection for their identification is facultative” (Round 2)?” | 0 | 1 | 5 | 9 | 93.3 | 0 | 0 | 5 | 10 | 100 |
| Do you agree that Step 21 is “Dissection of the posterior and lateral aspects of the adrenal gland *en bloc* with periadrenal fat”? | 0 | 0 | 4 | 11 | 100 | n/a | n/a | n/a | n/a | n/a |
| Do you agree that Step 22 is “Dissection of the superior aspect of the adrenal gland *en bloc* with periadrenal fat”? | 0 | 0 | 4 | 11 | 100 | n/a | n/a | n/a | n/a | n/a |
| Do you agree that in case of large adrenal lesions, RTLA Phase 4- Dissection of adrenal gland may be performed prior to Phase 3- Dissection of the main adrenal vein? | n/a | n/a | n/a | n/a | n/a | 0 | 1 | 6 | 8 | 93.3 |
| **Phase 5**  Do you agree that RTLA Phase 5 is “Extraction and disassembling”? | 0 | 0 | 4 | 11 | 100 | n/a | n/a | n/a | n/a | n/a |
|  |  |  |  |  |  |  |  |  |  |  |
| Do you agree that Step 23 is “Specimen retrieval in a plastic bag”? | 0 | 0 | 2 | 13 | 100 | n/a | n/a | n/a | n/a | n/a |
| Do you agree that Step 24 is “Verification of haemostasis”? | 0 | 1 | 3 | 11 | 93.3 | 0 | 0 | 6 | 9 | 100 |
| Do you agree that Step 25 is “Drainage insertion” and that it is facultative? | 0 | 4 | 5 | 6 | **73.3** | 0 | 1 | 9 | 5 | 93.3 |
| Do you agree that Step 26 is “Trocar removal”? | 0 | 0 | 4 | 11 | 100 | n/a | n/a | n/a | n/a | n/a |
| Do you agree that Step 27 is “Port site closure”? | 0 | 0 | 4 | 11 | 100 | n/a | n/a | n/a | n/a | n/a |
| **Phase 6**  Do you agree that RTLA Phase 6 is " Other intervention"? | 0 | 1 | 7 | 7 | 93.3 | 0 | 1 | 7 | 7 | 93.3 |

**Table S1.** **Number of responses (strongly disagree/disagree/agree/strongly agree) and percentage of agreement to statements regarding the proposed RTLA ontology during the two Delphi rounds.**

SD = strongly disagree, D = disagree, A = agree, SA = strongly agree, %A = percentage of agreement, IVC= Inferior Vena Cava. n/a= not applicable (either due to 100% agreement in the first round or due to addition in the second round)

| **Statements** | **Round 1 (*n*=15)** | | | | | **Round 2 (*n*=15)** | | | | |
| --- | --- | --- | --- | --- | --- | --- | --- | --- | --- | --- |
|  | **SD** | **D** | **A** | **SA** | **A%** | **SD** | **D** | **A** | **SA** | **A%** |
| **Phase 1**  Do you agree that LTLA Phase 1 is “Preparation”? | 0 | 0 | 3 | 12 | 100 | n/a | n/a | n/a | n/a | n/a |
|  |  |  |  |  |  |  |  |  |  |  |
| Do you agree that Step 0 is “Null step” and that it is facultative? | 0 | 2 | 4 | 9 | 86.7 | 0 | 0 | 5 | 10 | 100 |
| Concerning the facultative step 0, do you agree that it can also be recorded in each subsequent phase of LTLA? | 0 | 3 | 5 | 7 | 80 | 0 | 0 | 6 | 9 | 100 |
| Do you agree that Step 1 is “Trocar positioning”? | 0 | 1 | 3 | 11 | 93.3 | 0 | 0 | 5 | 10 | 100 |
| Do you agree that Step 2 is “Abdominal cavity exploration”? | 0 | 1 | 4 | 10 | 93.3 | 0 | 0 | 6 | 9 | 100 |
| **Phase 2**  Do you agree that LTLA Phase 2 is “Exposure”? | 0 | 0 | 2 | 13 | 100 | n/a | n/a | n/a | n/a | n/a |
|  |  |  |  |  |  |  |  |  |  |  |
| Do you agree that Step 3 is “Adhaesiolysis” and that it is facultative? | 0 | 1 | 5 | 9 | 93.3 | 0 | 0 | 7 | 8 | 100 |
| Do you agree that Step 4 is “Dissection of the left colonic flexure”? | 0 | 0 | 4 | 11 | 100 | n/a | n/a | n/a | n/a | n/a |
| Do you agree that Step 5 is “Dissection of splenoparietal ligament and mobilization of the tail of the pancreas until visualization of gastric fundus and the left diaphragmatic pillar (Jinnai manoeuvre)"? | 0 | 1 | 1 | 13 | 93.3 | 0 | 0 | 4 | 11 | 100 |
| Do you agree that Step 6 is “Haemostasis” and that it is facultative? | 0 | 2 | 5 | 8 | 86.7 | 0 | 0 | 9 | 6 | 100 |
| Concerning the facultative Step 6, do you agree that it can also be recorded in each subsequent phase of RTLA? | 0 | 0 | 7 | 8 | 100 | n/a | n/a | n/a | n/a | n/a |
| Do you agree that Step 7 is “Irrigation- aspiration” and that it is facultative? | 0 | 1 | 7 | 7 | 93.3 | 0 | 1 | 7 | 7 | 93.3 |
| Concerning the facultative Step 7, do you agree that it can also be recorded in each subsequent phase of LTLA? | 0 | 1 | 8 | 6 | 93.3 | 0 | 0 | 8 | 7 | 100 |
| **Phase 3**  Do you agree that LTLA Phase 3 is “Dissection of the main adrenal vein”? | 0 | 2 | 4 | 9 | 86.7 | 0 | 0 | 9 | 6 | 100 |
|  |  |  |  |  |  |  |  |  |  |  |
| Do you agree that Step 8 is “Opening of Gerota’s fascia at the medial aspect of the left adrenal gland”? | 0 | 2 | 3 | 100 | 86.7 | 0 | 0 | 8 | 7 | 100 |
| Do you agree that Step 9 is “Identification of the left renal vein”? | 0 | 1 | 3 | 11 | 93.3 | 0 | 0 | 6 | 9 | 100 |
| Do you agree that Step 10 is “Identification of the left inferior phrenic vein" and that is facultative? | 0 | 0 | 5 | 10 | 100 | n/a | n/a | n/a | n/a | n/a |
| Do you agree that Step 11 is “Identification of the left main adrenal vein”? | 0 | 0 | 3 | 11 | 100 | n/a | n/a | n/a | n/a | n/a |
| Do you agree that Step 12 is “Ligation and division of the left main adrenal vein (Round 1) / Sealing and division of the left main adrenal vein (Round 2)”? | 0 | 0 | 3 | 12 | 100 | 0 | 0 | 7 | 8 | 100 |
| Do you agree that Step 13 is “Identification of an accessory adrenal vein” and that is facultative? | 0 | 0 | 6 | 9 | 100 | n/a | n/a | n/a | n/a | n/a |
| Do you agree that Step 14 is “Ligation and division of the accessory adrenal vein” and that it is facultative (Round 1) / Sealing and division of the accessory adrenal vein” and that it is facultative (Round 2)”? | 0 | 0 | 6 | 9 | 100 | 0 | 0 | 7 | 8 | 100 |
| Do you agree that Step 15 is “Identification of adrenal arteries” and that it is facultative? | 0 | 0 | 7 | 8 | 100 | n/a | n/a | n/a | n/a | n/a |
| Concerning the facultative Step 15, do you agree that it can also be recorded in the subsequent phase of LTLA? | 0 | 0 | 9 | 6 | 100 | n/a | n/a | n/a | n/a | n/a |
| Do you agree that Step 16 is “Ligation and division of adrenal arteries” and that it is facultative (Round 1) / Sealing and division of adrenal arteries” and that it is facultative (Round 2)?” | 0 | 2 | 7 | 6 | 86.7 | 0 | 0 | 9 | 6 | 100 |
| Concerning the facultative Step 16, do you agree that it can also be recorded in the subsequent phase of LTLA? | 0 | 0 | 8 | 7 | 100 | 0 | 0 | 8 | 7 | 100 |
| **Phase 4**  Do you agree that LTLA Phase 4 is “Dissection of the adrenal gland”? | 0 | 1 | 2 | 12 | 93.3 | 0 | 0 | 7 | 8 | 100 |
|  |  |  |  |  |  |  |  |  |  |  |
| Do you agree that Step 17 is “Dissection of the adrenal gland from the upper pole of the kidney *en* *bloc* with periadrenal fat”? | 0 | 0 | 4 | 11 | 100 | n/a | n/a | n/a | n/a | n/a |
| Do you agree that Step 18 is “Identification and preservation of superior pole renal arteries” and that it is facultative (Round 1) / “Identification and preservation of superior pole renal arteries and that preservation of the vessels is compulsory, but dissection for their identification is facultative” (Round 2)?” | 0 | 1 | 3 | 11 | 93.3 | 0 | 0 | 8 | 7 | 100 |
| Do you agree that Step 19 is “Dissection of the posterior and lateral aspects of the adrenal gland *en bloc* with periadrenal fat”? | 0 | 0 | 4 | 11 | 100 | n/a | n/a | n/a | n/a | n/a |
| Do you agree that Step 20 is “Dissection of the superior aspect of the adrenal gland *en bloc* with periadrenal fat”? | 0 | 0 | 4 | 11 | 100 | n/a | n/a | n/a | n/a | n/a |
| Do you agree that in case of large adrenal lesions, LTLA Phase 4- Dissection of adrenal gland may be performed prior to Phase 3- Dissection of the main adrenal vein? | n/a | n/a | n/a | n/a | n/a | 0 | 1 | 6 | 8 | 93.3 |
| **Phase 5**  Do you agree that LTLA Phase 5 is “Extraction and disassembling”? | 0 | 0 | 6 | 9 | 100 | n/a | n/a | n/a | n/a | n/a |
|  |  |  |  |  |  |  |  |  |  |  |
| Do you agree that Step 21 is “Specimen retrieval in a plastic bag”? | 0 | 0 | 3 | 12 | 100 | n/a | n/a | n/a | n/a | n/a |
| Do you agree that Step 22 is “Verification of haemostasis”? | 0 | 1 | 3 | 11 | 93.3 | 0 | 0 | 6 | 9 | 100 |
| Do you agree that Step 23 is “Drainage insertion” and that it is facultative? | 0 | 3 | 6 | 6 | 80 | 0 | 2 | 7 | 6 | 86.7 |
| Do you agree that Step 24 is “Trocar removal”? | 0 | 0 | 4 | 11 | 100 | n/a | n/a | n/a | n/a | n/a |
| Do you agree that Step 25 is “Port site closure”? | 0 | 0 | 3 | 12 | 100 | n/a | n/a | n/a | n/a | n/a |
| **Phase 6**  Do you agree that LTLA Phase 6 is " Other intervention"? | 0 | 1 | 5 | 9 | 93.3 | 0 | 0 | 7 | 8 | 100 |

**Table S2**. **Number of responses (strongly disagree/disagree/agree/strongly agree) and percentage of agreement to statements regarding the proposed LTLA ontology during the two Delphi rounds.**

SD = strongly disagree, D = disagree, A = agree, SA = strongly agree, % A = percentage of agreement

n/a= not applicable (either due to 100% agreement in the first round or due to addition in the second round)

References

1.	Bellantone R, De Crea C. Surgical procedure: adrenalectomy–indications, operative techniques and management of complications. In: *Endocrine Surgery Comprehensive Board Exam Guide*. Springer, 2022; 575-614.

2.	Meireles OR, Rosman G, Altieri MS, Carin L, Hager G, Madani A, Padoy N, Pugh CM, Sylla P, Ward TM, Hashimoto DA, Groups SVAfAW. SAGES consensus recommendations on an annotation framework for surgical video. *Surg Endosc* 2021;**35**(9): 4918-4929.

3.	Lavanchy JL, Gonzalez C, Kassem H, Nett PC, Mutter D, Padoy N. Proposal and multicentric validation of a laparoscopic Roux-en-Y gastric bypass surgery ontology. *Surg Endosc* 2023;**37**(3): 2070-2077.

4.	Powell C. The Delphi technique: myths and realities. *J Adv Nurs* 2003;**41**(4): 376-382.

5.	Okoli C, Pawlowski SD. The Delphi method as a research tool: an example, design considerations and applications. *Information & Management* 2004;**42**(1): 15-29.

6.	Paliwoda SJ. Predicting the Future Using Delphi. *Management Decision* 1983;**21**(1): 31-38.

7.	Gibaud B, Forestier G, Feldmann C, Ferrigno G, Goncalves P, Haidegger T, Julliard C, Katic D, Kenngott H, Maier-Hein L, Marz K, de Momi E, Nagy DA, Nakawala H, Neumann J, Neumuth T, Rojas Balderrama J, Speidel S, Wagner M, Jannin P. Toward a standard ontology of surgical process models. *Int J Comput Assist Radiol Surg* 2018;**13**(9): 1397-1408.

8.	Garrow CR, Kowalewski KF, Li L, Wagner M, Schmidt MW, Engelhardt S, Hashimoto DA, Kenngott HG, Bodenstedt S, Speidel S, Muller-Stich BP, Nickel F. Machine Learning for Surgical Phase Recognition: A Systematic Review. *Ann Surg* 2021;**273**(4): 684-693.

9.	Komatsu M, Kitaguchi D, Yura M, Takeshita N, Yoshida M, Yamaguchi M, Kondo H, Kinoshita T, Ito M. Automatic surgical phase recognition-based skill assessment in laparoscopic distal gastrectomy using multicenter videos. *Gastric Cancer* 2024;**27**(1): 187-196.

10.	Kolbinger FR, Bodenstedt S, Carstens M, Leger S, Krell S, Rinner FM, Nielen TP, Kirchberg J, Fritzmann J, Weitz J, Distler M, Speidel S. Artificial Intelligence for context-aware surgical guidance in complex robot-assisted oncological procedures: An exploratory feasibility study. *Eur J Surg Oncol* 2023: 106996.

11.	Mihai R, Donatini G, Vidal O, Brunaud L. Volume-outcome correlation in adrenal surgery-an ESES consensus statement. *Langenbecks Arch Surg* 2019;**404**(7): 795-806.

12.	Raffaelli M, De Crea C, Bellantone R. Laparoscopic adrenalectomy. *Gland Surg* 2019;**8**(Suppl 1): S41-S52.

13.	Fassnacht M, Assie G, Baudin E, Eisenhofer G, de la Fouchardiere C, Haak HR, de Krijger R, Porpiglia F, Terzolo M, Berruti A, clinicalguidelines@esmo.org EGCEa. Adrenocortical carcinomas and malignant phaeochromocytomas: ESMO-EURACAN Clinical Practice Guidelines for diagnosis, treatment and follow-up. *Ann Oncol* 2020;**31**(11): 1476-1490.

14.	Garcia-Carbonero R, Matute Teresa F, Mercader-Cidoncha E, Mitjavila-Casanovas M, Robledo M, Tena I, Alvarez-Escola C, Aristegui M, Bella-Cueto MR, Ferrer-Albiach C, Hanzu FA. Multidisciplinary practice guidelines for the diagnosis, genetic counseling and treatment of pheochromocytomas and paragangliomas. *Clin Transl Oncol* 2021;**23**(10): 1995-2019.

15.	Akins RB, Tolson H, Cole BR. Stability of response characteristics of a Delphi panel: application of bootstrap data expansion. *BMC Med Res Methodol* 2005;**5**: 37.

16.	McPherson S, Reese C, Wendler MC. Methodology Update: Delphi Studies. *Nurs Res* 2018;**67**(5): 404-410.

17.	Murphy MK, Black NA, Lamping DL, McKee CM, Sanderson CF, Askham J, Marteau T. Consensus development methods, and their use in clinical guideline development. *Health Technol Assess* 1998;**2**(3): i-iv, 1-88.

18.	Hasson F, Keeney S, McKenna H. Research guidelines for the Delphi survey technique. *J Adv Nurs* 2000;**32**(4): 1008-1015.

19.	Diamond IR, Grant RC, Feldman BM, Pencharz PB, Ling SC, Moore AM, Wales PW. Defining consensus: a systematic review recommends methodologic criteria for reporting of Delphi studies. *J Clin Epidemiol* 2014;**67**(4): 401-409.

20.	Cesmebasi A, Du Plessis M, Iannatuono M, Shah S, Tubbs RS, Loukas M. A review of the anatomy and clinical significance of adrenal veins. *Clin Anat* 2014;**27**(8): 1253-1263.

21.	Scholten A, Cisco RM, Vriens MR, Shen WT, Duh QY. Variant adrenal venous anatomy in 546 laparoscopic adrenalectomies. *JAMA Surg* 2013;**148**(4): 378-383.

22.	Brook NR, Dell'Oglio P, Barod R, Collins J, Mottrie A. Comprehensive training in robotic surgery. *Curr Opin Urol* 2019;**29**(1): 1-9.

23.	Seeliger B, Collins T, Marescaux J. Implemented Imaging and Artificial Intelligence. In: *The Foundation and Art of Robotic Surgery*, Giulianotti PC, Benedetti E, Mangano A (eds). McGraw-Hill: New York, NY, 2024.

24.	Marescaux J, Rubino F, Arenas M, Mutter D, Soler L. Augmented-reality-assisted laparoscopic adrenalectomy. *JAMA* 2004;**292**(18): 2214-2215.

25.	Neumann J, Uciteli A, Meschke T, Bieck R, Franke S, Herre H, Neumuth T. Ontology-based surgical workflow recognition and prediction. *J Biomed Inform* 2022;**136**: 104240.

26.	Farinha R, Breda A, Porter J, Mottrie A, Van Cleynenbreugel B, Vander Sloten J, Mottaran A, Gallagher AG, Group RA-DS. International Expert Consensus on Metric-based Characterization of Robot-assisted Partial Nephrectomy. *Eur Urol Focus* 2023;**9**(2): 388-395.
